# Causal relationships between migraine and microstructural white matter: a Mendelian randomization study

**DOI:** 10.1186/s10194-023-01550-z

**Published:** 2023-02-16

**Authors:** Lei Zhao, Wenhui Zhao, International Headache Genetics Consortium (IHGC), Verneri Anttila, Ville Artto, Andrea C. Belin, Anna Bjornsdottir, Gyda Bjornsdottir, Dorret I. Boomsma, Sigrid Børte, Mona A. Chalmer, Daniel I. Chasman, Bru Cormand, Ester Cuenca-Leon, George Davey-Smith, Irene de Boer, Martin Dichgans, Tonu Esko, Tobias Freilinger, Padhraig Gormley, Lyn R. Griffiths, Eija Hämäläinen, Thomas F. Hansen, Aster V. E. Harder, Heidi Hautakangas, Marjo Hiekkala, Maria G. Hrafnsdottir, M. Arfan Ikram, Marjo-Riitta Järvelin, Risto Kajanne, Mikko Kallela, Jaakko Kaprio, Mari Kaunisto, Lisette J. A. Kogelman, Espen S. Kristoffersen, Christian Kubisch, Mitja Kurki, Tobias Kurth, Lenore Launer, Terho Lehtimäki, Davor Lessel, Lannie Ligthart, Sigurdur H. Magnusson, Rainer Malik, Bertram Müller-Myhsok, Carrie Northover, Dale R. Nyholt, Jes Olesen, Aarno Palotie, Priit Palta, Linda M. Pedersen, Nancy Pedersen, Matti Pirinen, Danielle Posthuma, Patricia Pozo-Rosich, Alice Pressman, Olli Raitakari, Caroline Ran, Gudrun R. Sigurdardottir, Hreinn Stefansson, Kari Stefansson, Olafur A. Sveinsson, Gisela M. Terwindt, Thorgeir E. Thorgeirsson, Arn M. J. M. van den Maagdenberg, Cornelia van Duijn, Maija Wessman, Bendik S. Winsvold, John-Anker Zwart, Jin Cao, Yiheng Tu

**Affiliations:** 1 CAS Key Laboratory of Mental Health, Institute of Psychology, Chinese Academy of Sciences, Beijing, China; 2 Department of Psychology, University of Chinese Academy of Sciences, Beijing, China; 3 School of Life Sciences, Beijing University of Chinese Medicine, Beijing, China

**Keywords:** Migraine, Microstructural white matter, Imaging-derived phenotype, Causal association, Mendelian randomization

## Abstract

**Background:**

Migraine is a disabling neurological disorder with the pathophysiology yet to be understood. The microstructural alteration in brain white matter (WM) has been suggested to be related to migraine in recent studies, but these evidence are observational essentially and cannot infer a causal relationship. The present study aims to reveal the causal relationship between migraine and microstructural WM using genetic data and Mendelian randomization (MR).

**Methods:**

We collected the Genome-wide association study (GWAS) summary statistics of migraine (48,975 cases / 550,381 controls) and 360 WM imaging-derived phenotypes (IDPs) (31,356 samples) that were used to measure microstructural WM. Based on instrumental variables (IVs) selected from the GWAS summary statistics, we conducted bidirectional two-sample MR analyses to infer bidirectional causal associations between migraine and microstructural WM. In forward MR analysis, we inferred the causal effect of microstructural WM on migraine by reporting the odds ratio (OR) that quantified the risk change of migraine for per 1 standard deviation (SD) increase of IDPs. In reverse MR analysis, we inferred the causal effect of migraine on microstructural WM by reporting the *β* value that represented SDs of changes in IDPs were caused by migraine.

**Results:**

Three WM IDPs showed significant causal associations (*p* < 3.29 × 10^− 4^, Bonferroni correction) with migraine and were proved to be reliable via sensitivity analysis. The mode of anisotropy (MO) of left inferior fronto-occipital fasciculus (OR = 1.76, *p* = 6.46 × 10^− 5^) and orientation dispersion index (OD) of right posterior thalamic radiation (OR = 0.78, *p* = 1.86 × 10^− 4^) exerted significant causal effects on migraine. Migraine exerted a significant causal effect on the OD of left superior cerebellar peduncle (*β* = − 0.09, *p* = 2.78 × 10^− 4^).

**Conclusions:**

Our findings provided genetic evidence for the causal relationships between migraine and microstructural WM, bringing new insights into brain structure for the development and experience of migraine.

**Supplementary Information:**

The online version contains supplementary material available at 10.1186/s10194-023-01550-z.

## Background

Migraine is a highly prevalent and disabling neurological disorder affecting approximately 14.4% of the global population [1]. It is primarily characterized by attacks of headache and is also accompanied by associated symptoms including nausea, vomiting, and hypersensitivity to light and sound [2]. Despite being a major source of disability worldwide, the pathophysiology of migraine remains poorly understood and expects further exploration [3]. Increasing evidence from functional magnetic resonance imaging (fMRI) studies has shown widespread functional abnormalities in cortical and subcortical regions during different phases of migraine [4, 5]. In association with functional alterations, morphometric techniques of structural MRI (sMRI), such as voxel-based morphometry, have shown brain grey matter structural abnormalities in patients with migraine [6, 7].

In addition to brain grey matter abnormalities, neuroimaging studies using diffusion-weighted MRI (dMRI) also examined the microstructural alterations in brain white matter (WM) and demonstrated abnormalities in widespread WM tracts in migraine patients [8, 9]. For instance, a dMRI study demonstrated that migraine patients showed alterations in the WM fibers connecting the thalamus, frontal lobes, and occipital lobes using both tract-based spatial statistics (TBSS) and probabilistic tractography analyses [10]. Another study identified that the WM cerebellar tracts also showed significant alterations in migraine patients [11]. Moreover, the WM tracts that connect the thalamus and cerebral cortex were altered in migraine patients and showed a significant correlation with the years lived with migraine [12]. Thus, the accumulating evidence has suggested that migraine is associated with the changes of microstructural WM, but most of them are observational essentially and whether the relationships are causal is unclear.

Mendelian randomization (MR) is widely used in epidemiological studies by introducing genetic variants as instruments to draw causal inference from observational data [13, 14]. MR minimizes the issues of reverse causality and confounders in observational data because genetic variants are innate and relatively independent of self-selected behaviors [15, 16]. Large-scale genome-wide association studies (GWAS) on both brain imaging-derived phenotypes (IDPs) and neuropsychiatric disorders provide opportunities to explore the causality between exposure (e.g., brain IDPs) and outcome (e.g., diseases) [17]. Using MR, a recent study explored the causal effects of subcortical regions and intracranial volume (ICV) on migraine [18]. MR results demonstrated a possible causal relationship between smaller ICV, hippocampal and ventral diencephalon volume and increased migraine risk, while reverse MR showed causality between increased migraine risk and a larger volume of the amygdala. However, the bidirectional causal associations between migraine and microstructural WM remain unclear.

In the present study, we aim to investigate the causal effect of microstructural WM on migraine and vice versa based on the genetic variants of WM IDPs and migraine. First, we searched and collected the GWAS summary statistics of migraine and 360 WM IDPs to obtain the genetic variants. To reduce multiple testing on subsequent MR analysis, the linkage disequilibrium (LD)-score regression was then performed on the summary statistics of each ‘IDP- migraine’ pair to screen the WM IDPs genetically correlated with migraine. We then used two-sample MR analysis to infer the bidirectional causal associations between migraine and microstructural WM based on the GWAS summary statistics of migraine and the screened WM IDPs. In forward MR analysis, genetic instrumental variables (IVs) were filtered from the GWAS summary statistics of WM IDPs to estimate the odds ratio (OR) that quantified the risk change of migraine for per 1 standard deviation (SD) increase of IDPs. In reverse MR analysis, genetic IVs were filtered from the GWAS summary statistics of migraine to estimate the *β* value that represented how many SDs of changes in IDPs were caused by migraine. Finally, sensitivity analysis was conducted to exclude spurious causal associations in MR results.

## Methods

### Datasets

In the present study, the GWAS summary statistics of migraine were obtained from a recent GWAS meta-analysis study [19]. Because of the privacy protection for the participants of the 23andMe cohort, the GWAS summary statistics used in this study excluded the samples from the 23andMe cohort. The GWAS summary statistics were obtained from 599,356 individuals (N_case_ = 48,975 and N_control_ = 550,381) of European ancestry from 24 cohorts.

With regard to microstructural WM, GWAS summary statistics of WM IDPs were obtained from a genetic study on IDPs of the brain (https://open.win.ox.ac.uk/ukbiobank/big40) [20]. The sample of GWAS consists of 31,356 individuals of European ancestry from UK Biobank, a large-scale biomedical database containing genetic and health information from over 500,000 UK participants aged between 40 and 69 at recruitment and aiming to improve human health and modern medicine.

To be specific, UK Biobank has collected both genetic and multimodal brain imaging data from around 50,000 individuals. In UK Biobank, MRI scanning was performed using a 3.0-T MRI Scanner (Siemens Skyra, Siemens Healthcare, Erlangen, Germany) with a 32-channel radiofrequency receiver head coil. The dMRI data were acquired using a standard (monopolar) spin-echo echo-planar imaging sequence with 5 baseline images (b = 0 seconds mm^− 2^), 50 diffusion-weighted images with b = 1000 seconds mm^− 2^, and 50 diffusion-weighted images with b = 2000 s mm^− 2^, along with following imaging parameters: TR/TE = 3600/92 ms, FOV = 104 × 104 mm^2^, resolution = 2x2x2 mm^3^, slice number = 72, slice thickness = 2 mm, and multislice acceleration = 3. Based on the dMRI data, UK Biobank characterized the microstructure of WM via two complementary analyses, i.e., TBSS and probabilistic tractography analyses. TBSS analysis characterized 48 WM tracts using the brain atlas provided by Johns Hopkins University [21]. Probabilistic tractography analysis characterized 27 WM tracts using the brain atlas defined by AutoPtx [22]. Both TBSS and probabilistic tractography analyses report 6 measurements within multiple tract regions using the diffusion tensor imaging (DTI) fitting tool DTIFIT and Neurite Orientation Dispersion and Density Imaging (NODDI) fitting tool AMICO [23], including fractional anisotropy (FA), mode of anisotropy (MO), mean diffusivity (MD), intracellular volume fraction (ICVF), orientation dispersion index (OD), and isotropic or free water volume fraction (ISOVF).

Consistent with a recent study that estimated the bidirectional casual associations between microstructural WM and psychiatric disorders [17], we excluded 15 of 27 probabilistic tractography-based WM tracts that were also characterized by TBSS analysis, resulting in a total of 60 WM tracts (12 probabilistic tractography and 48 TBSS) in the following analyses. Finally, 360 WM IDPs (60 WM tracts × 6 measurements) were included in the present study, with each IDP presenting the mean (TBSS-based tracts) or weighted-mean value (probabilistic tractography-based tracts) of a measurement within a WM tract. Details (e.g., the ID and name) of these IDPs are available in Additional file 1: Appendix S1.

### Genetic correlation analysis

A feasible strategy to perform multi-pair formal MR analyses needs to explore potential genetic correlations in the first step. If two heritable traits are causally related, there should be a genetic correlation between them [24]. This strategy has been used in previous studies to reduce multiple testing [17, 25]. Therefore, we first performed genetic correlation analysis before MR analysis to screen the WM IDPs that were genetically associated with migraine. Based on the GWAS summary statistics of migraine and 360 WM IDPs, genetic correlation analysis was performed on each ‘IDP- migraine’ pair using LD-score regression [26]. The software and protocol to run LD-score regression are publicly available (https://github.com/bulik/ldsc). As recommended by the developers, Single Nucleotide Polymorphisms (SNPs) were filtered using the HapMap-3 reference panel with a 1000 Genomes EUR minor allele frequency (MAF) > 1% (https://alkesgroup.broadinstitute.org/LDSCORE/w_hm3.snplist.bz2) and major histocompatibility complex region was further excluded due to unusual LD within this region. The precalculated LD scores and weights were obtained from the Broad institute (https://data.broadinstitute.org/alkesgroup/LDSCORE/eur_w_ld_chr.tar.bz2). A two-tailed *p* < 0.05 was set as the cutoff to preserve IDPs for subsequent MR analysis.

### Two-sample MR analysis

**Fig. 1:**
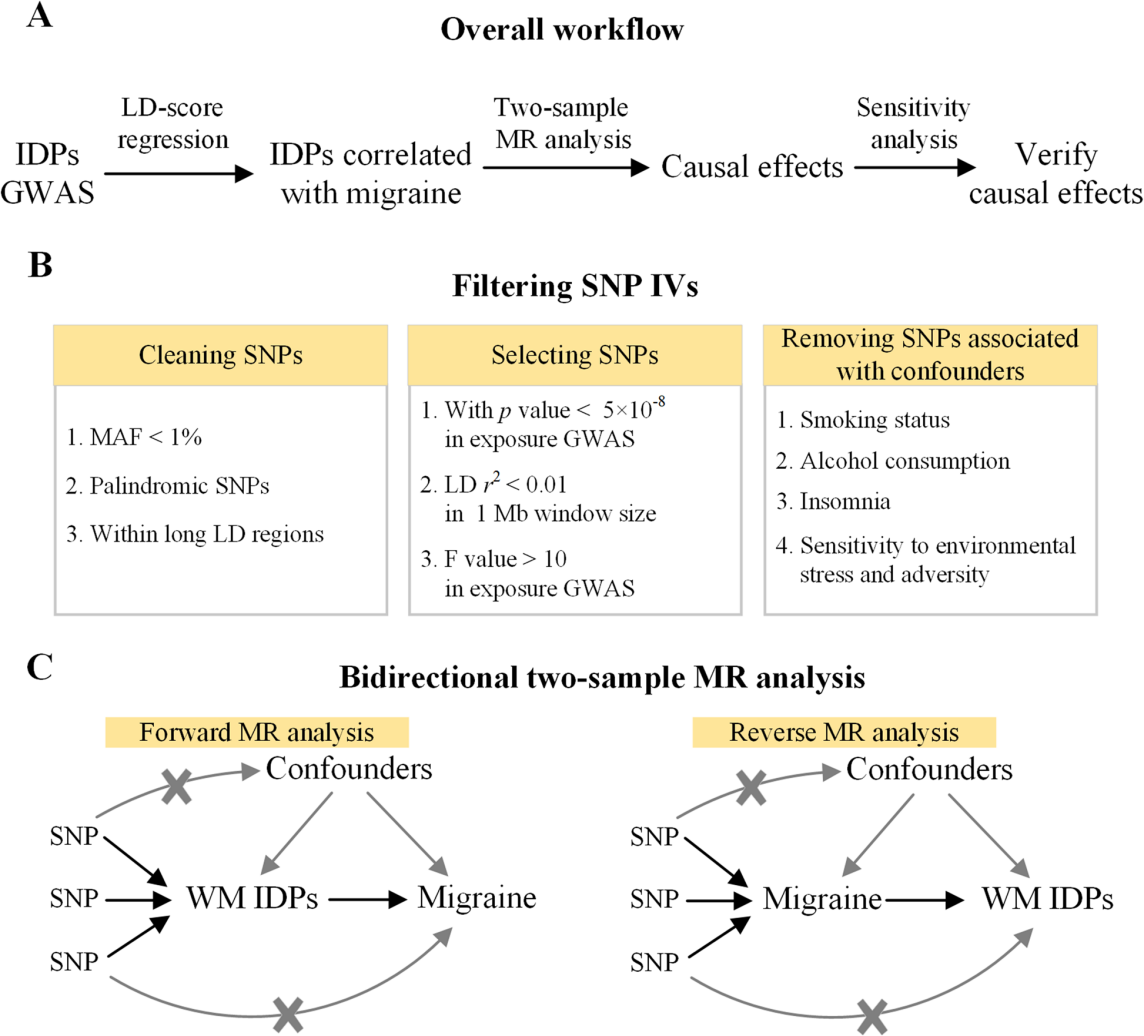
Flowchart of the study. **A** Overall workflow of the causal inference between migraine and microstructural WM. **B** Filtering SNP IVs for two-sample MR analysis. **C** Simplified illustration of Mendelian randomization. Abbreviations: IDPs, imaging-derived phenotypes; GWAS, genome-wide association study; LD, linkage disequilibrium; MAF, minor allele frequency; MR, Mendelian randomization; SNP, single nucleotide polymorphism; WM, white matter

As shown in the overall workflow (Fig. 1A), MR analysis was performed on the WM IDPs that had shown significant genetic correlations with migraine. For migraine and each IDP, genetic variants with MAF < 1% were first removed from GWAS summary statistics. To avoid ambiguous harmonization of effect allele caused by strand flipping issues, the palindromic SNPs (i.e., alleles are A/T or G/C) with MAF close to 50% were further removed [27]. We also removed the SNPs within long LD regions to ensure the independence of SNP IVs because the correlation between SNP IVs may bias the MR estimation [28].

After cleaning the above SNPs, we selected the IVs for MR analysis from GWAS summary statistics. Following the hypothesis that IVs should be strongly associated with exposure, the SNP IVs were first extracted from the GWAS summary statistics of exposure with a genome-wide significance threshold of *p* < 5 × 10^− 8^. Then, a clumping algorithm was used to select independent SNP IVs with a *r*^2^ threshold of 0.01 in a window size of 1 Mb. To reduce weak instrument bias, only the SNPs with a F value > 10 in GWAS of exposure were considered as potential IVs [29]. The SNPs that were directly associated with outcomes or confounding factors were removed from IVs to exclude potential horizontal pleiotropy. The SNPs significantly associated with the confounders were acquired from the NHGRI-EBI GWAS catalog (https://www.ebi.ac.uk/gwas/docs/file-downloads). Four confounders, including smoking status, alcohol consumption, insomnia, and sensitivity to environmental stress and adversity, were considered in the present study, because these traits are common risk factors for both migraine and brain structures [30–32]. Finally, the retained SNPs were used as the IVs of MR analysis (Fig. 1B).

In the present study, we performed a bidirectional two-sample MR analysis using TwoSampleMR R package (https://mrcieu.github.io/TwoSampleMR) [33] (Fig. 1C). The forward MR analysis was performed with each IDP as exposure and migraine as outcome. Conversely, the reverse MR analysis was performed with migraine as exposure and each IDP as outcome. We quantified the causal effects using OR and *β* values in forward and reverse MR analyses, respectively. Because WM IDPs have been standardized before conducting GWAS [20], OR represents the risk change of migraine for per 1 SD increase of IDPs and *β* value represents how many SD of changes in IDPs were caused by migraine.

The inverse-variance weighted (IVW) method with a random effects model was used for main MR analysis to evaluate the causal effect of exposure on outcome due to its high power to detect a causal effect [34, 35]. Consistent with previous studies, we employed four supplementary MR methods (weighted median, simple mode, weighted mode, and MR-Egger method) to assess the reliability of the main results [17, 36]. The Wald ratio method was used for the main MR analysis when only a single SNP IV was available [37]. The statistical results were corrected for multiple testing with Bonferroni across all genetically correlated ‘IDP- migraine’ pairs in both directions. Thus, the corrected significant threshold was set as two-tailed *p* < 3.29 × 10^− 4^ (0.05/76/2; 76 is the number of ‘IDP- migraine’ pairs screened out by abovementioned genetic correlation analysis, and 2 denotes both directions of MR analysis).

### Sensitivity analysis

The results of MR analysis were further verified by implementing a series of sensitivity analyses. First, MR-Egger regression and MR-PRESSO Global test were used to examine the presence of horizontal pleiotropy [38, 39]. Second, Cochran’s Q statistic was employed to complete the heterogeneity test on the effect among SNPs [40]. Third, leave-one-out (LOO) analysis was performed to assess if the causal association was dominated by a single SNP that had a large horizontal pleiotropy.

## Results

### Genetic correlations

In the present study, 76 of 360 WM IDPs showed significant genetic correlations (*p* < 0.05) with migraine. We further investigated the causal associations between these IDPs and migraine using two-sample MR analyses. The detailed results of genetic correlations are shown in Additional file 1: Appendix S2.

### The causal effects of WM IDPs on migraine

**Fig. 2:**
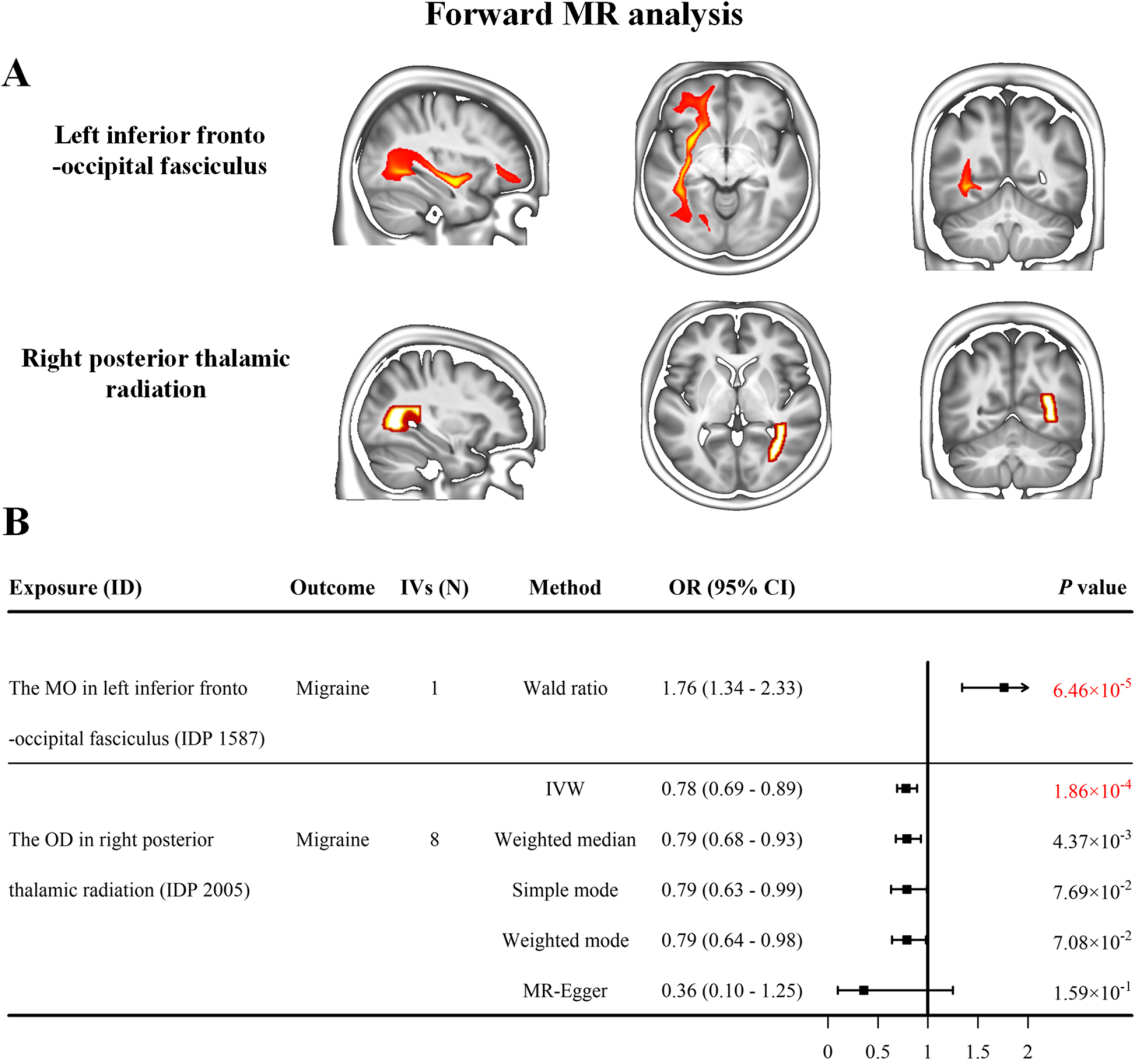
The results of the forward MR analysis. **A** The anatomical locations of the WM IDPs showing significant causal associations with migraine in the forward MR analysis. **B** The forest plot shows the results of causal estimates. Each box represents the effect (i.e., OR change) per 1 SD change in the respective IDP on migraine, and the error bars represent 95% CI. Arrows indicate that 95% CI exceeds the x axis. Significant causal correlation is defined as *p* value < 3.29 × 10^− 4^ (Bonferroni correction) and marked by red chroma. The IVW (if the number of IVs > 1) -derived or Wald ratio (if the number of IVs = 1) -derived results were regarded as main results. Abbreviations: CI, confidence interval; IDP, imaging-derived phenotypes; IVs, instrumental variables; IVW, inverse variance-weighted; MR, Mendelian randomization; OR, odds ratio

Two-sample MR analysis identified two WM IDPs (Fig. 2A) that exhibited significant causal effects on migraine in the forward MR analysis (*p* < 3.29e × 10^− 4^, Bonferroni correction). Specifically, the left inferior fronto-occipital fasciculus, a WM tract originates in the occipital and parietal lobes and terminates in the inferior frontal lobe, showed a causal effect on migraine (Fig. 2B), with 1 SD increase in the MO (IDP 1587) associated with 76% increased odds of migraine risk (Wald ratio-derived OR = 1.76, 95% confidence interval (CI) = [1.34, 2.33], *p* = 6.46 × 10^− 5^). Per 1 SD decrease of OD (IDP 2005) in right posterior thalamic radiation (Fig. 2B), a fiber connects the caudal parts of the thalamus and occipital lobe and parietal lobe, decreased the risk of migraine by 22% (IVW-derived OR = 0.78, 95% CI = [0.69, 0.89], *p* = 1.86 × 10^− 4^). The effect directions estimated by the other four supplementary MR methods were consistent with the primary IVW analyses.

### The causal effects of migraine on WM IDPs

**Fig. 3:**
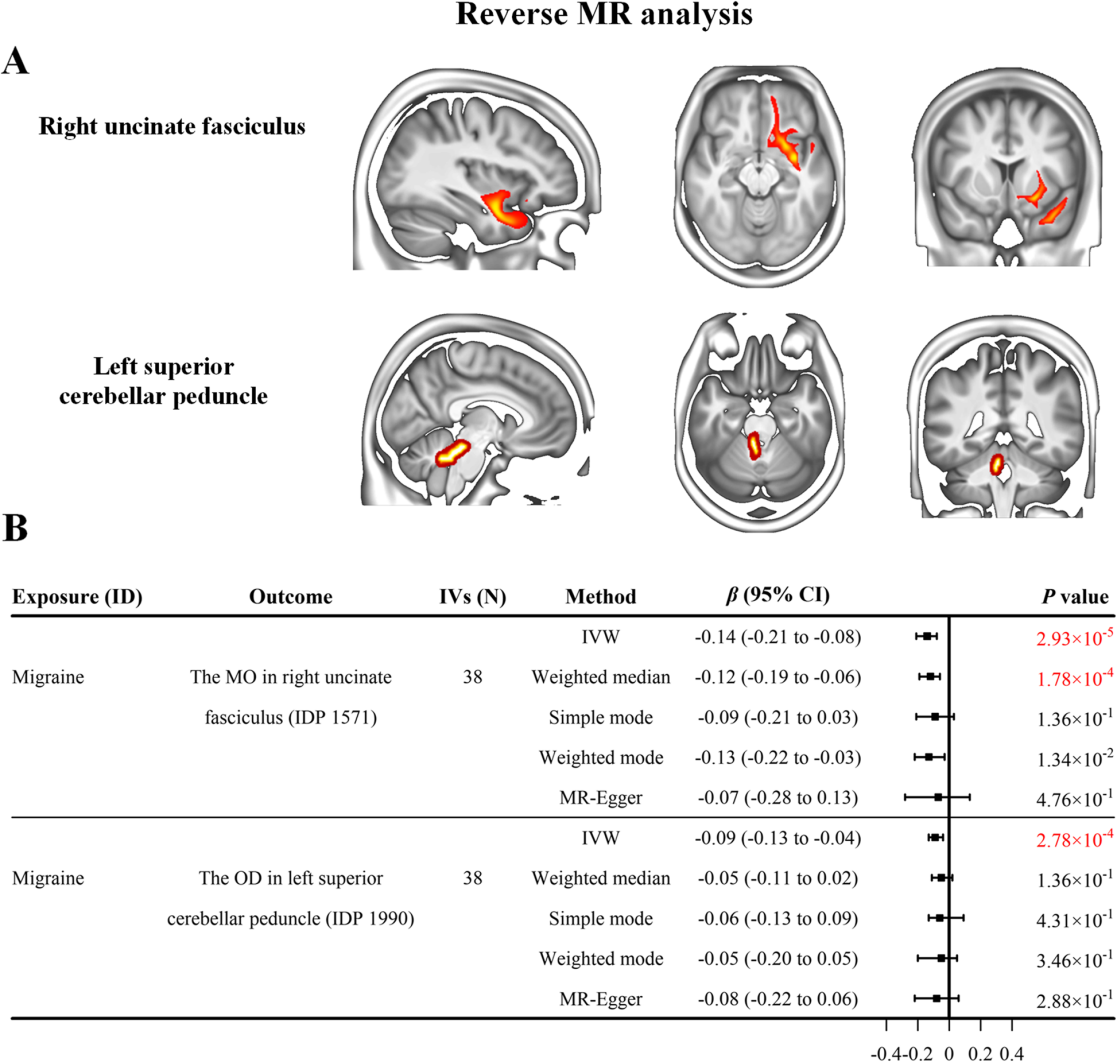
The results of the reverse MR analysis. **A** The anatomical locations of the IDPs showing significant causal relationship with migraine in the reverse MR analysis. **B** The forest plot shows the results of causal estimates. Each box represents the effect (i.e., SD change) of migraine on the respective IDP, and the error bars represent 95% CI. Significant causal correlation is defined as *p* value < 3.29 × 10^− 4^ (Bonferroni correction) and marked by red chroma. The IVW-derived results were regarded as main results. Abbreviations: CI, confidence interval; IDP, imaging-derived phenotypes; IVs, instrumental variables; IVW, inverse variance-weighted; MR, Mendelian randomization

The primary IVW analysis demonstrated that migraine exhibited significant causal effects on two WM IDPs (Fig. 3A) in the reverse MR analysis (*p* < 3.29 × 10^− 4^, Bonferroni correction). As shown in Fig. 3B, the MO of right uncinate fasciculus, a tract connects the temporal lobe and frontal lobe (IDP 1571), was significantly decreased by migraine (IVW-derived *β* = − 0.14, 95% CI = [− 0.21, − 0.08], *p* = 2.93 × 10^− 5^). Similar estimates as in the main IVW analysis but with lower magnitude (weighted median-derived *β* = − 0.12, 95% CI = [− 0.19, − 0.06], *p* = 1.78 × 10^− 4^) were obtained from the weighted median method. The OD of left superior cerebellar peduncle, a tract connects the cerebellum to the midbrain (IDP 1990), was also significantly decreased by migraine (IVW-derived *β* = − 0.09, 95% CI = [− 0.13, − 0.04], *p* = 2.78 × 10^− 4^). The effect directions estimated by the other four supplementary MR methods were consistent with the primary IVW analyses.

### Sensitivity analysis

**Fig. 4:**
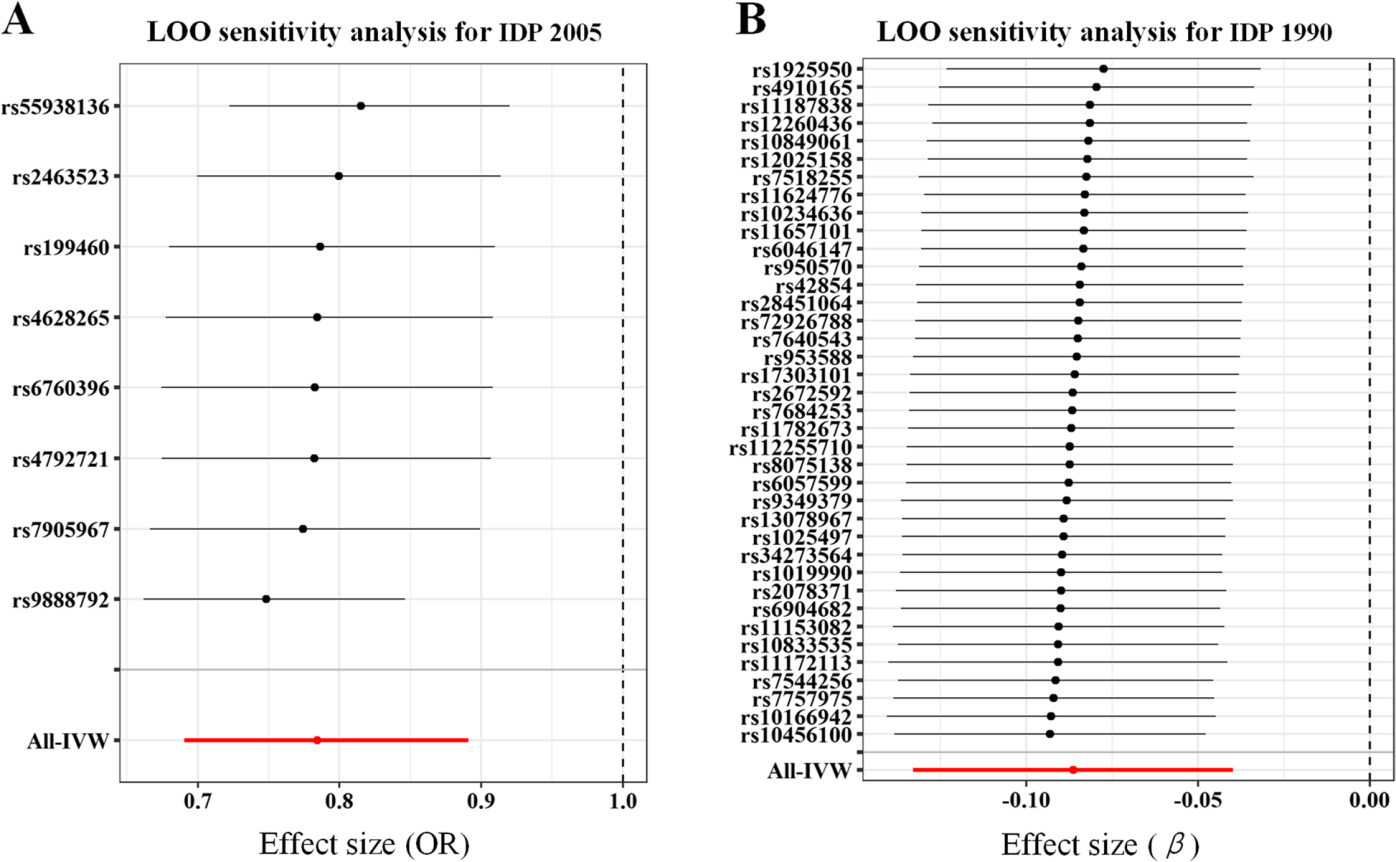
LOO sensitivity analysis. **A** Forward LOO sensitivity analysis for the OD in right posterior thalamic radiation (IDP 2005). **B** Reverse LOO sensitivity analysis for the OD in left superior cerebellar peduncle (IDP 1990). Each black point represents the IVW causal estimates after excluding the respective SNP from the analysis, and the error bars represent 95% CI. The red point represents the IVW causal estimates using all SNPs, and the error bars represent 95% CI. There is no dramatic change in causal estimates after excluding one particular SNP. Abbreviations: IDP, imaging-derived phenotypes; IVW, inverse variance-weighted; LOO, leave-one-out; OR, odds ratio

The causal effect of migraine on the MO of right uncinate fasciculus didn’t pass the test of horizontal pleiotropy and heterogeneity (Table 1). Specifically, though the MR-egger intercept was close to zero (*p* = 0.47), MR-PRESSO Global test detected significant horizontal pleiotropy (*p* < 0.05) in the causal effect of migraine on the MO of right uncinate fasciculus. The results of Cochran’s *Q*-test showed significant heterogeneity (*p* < 0.05) in the causal effect of migraine on the MO of right uncinate fasciculus. Thus, the causal effect of migraine on the MO of right uncinate fasciculus was excluded from subsequent sensitivity analysis. It is noted that the causal effect of the MO of left inferior fronto-occipital fasciculus on migraine was excluded from sensitivity analysis due to a single SNP. LOO analyses demonstrated that the causal estimates were not driven by any single SNP (Fig. 4).

**Table 1:** The results of sensitivity analysis

Exposure	Outcome	MR-egger regression	MR-PRESSO Global test	Cochran’s Q-test
The MO in left inferior fronto-occipital fasciculus (IDP 1587)	Migraine	NA	NA	NA
The OD in right posterior thalamic radiation (IDP 2005)	Migraine	Intercept = 0.04 *p* = 0.27	*RSS* = 10.96 *p* = 0.34	*Q*-value = 8.63 *p* = 0.28
Migraine	The MO in right uncinate fasciculus (IDP 1571)	Intercept = − 0.01 *p* = 0.47	*RSS* = 105.74 *p* < 1.00 × 10^− 3^	*Q*-value = 99.38 *p* = 1.29 × 10^− 7^
Migraine	The OD in left superior cerebellar peduncle (IDP 1990)	Intercept = − 0.01 *p* = 0.88	*RSS* = 49.10 *p* = 0.14	*Q*-value = 46.69 *p* = 0.13

*Abbreviations*: *IDPs* imaging-derived phenotypes, *MO* mode of anisotropy, *OD* orientation dispersion index, *RSS* residual sum of squares

The expected bias caused by sample overlap was quantified according to the method recommended by a previous technical study [41]. In the present study, migraine GWAS has a sample overlap proportion of 5.2% at most with WM IDPs GWAS. For each pair of causal association, the bias caused by sample overlap was less than 0.001, indicating that our causal estimates were less likely to be biased by sample overlaps.

## Discussion

The present study investigated the causal associations between migraine and microstructural WM based on genetic variants and two-sample MR. A total of 76 of 360 WM IDPs preliminarily showed genetic correlations with migraine, and 3 IDPs showed significant and reliable causal associations with migraine. In the forward MR analysis, an increase in the MO of left inferior fronto-occipital fasciculus and a decrease in the OD of right posterior thalamic radiation were found to elevate migraine risk. In the other direction, migraine decreased the OD of left superior cerebellar peduncle.

In our forward MR analysis, the risk of migraine was elevated by increased MO of left inferior fronto-occipital fasciculus, which is a long association fiber tract that structurally connects various parts of the occipital lobe with other cortical regions including the frontal, superior parietal, and temporo-basal areas [42–44]. As a tensor shape metric, the MO is sensitive to subtle differences in WM, particularly in crossing fibers, which exist in up to 90% of WM voxels in the brain [45, 46]. Increased MO of the inferior fronto-occipital fasciculus implies an abnormal information flow from the occipital lobe to other cortical regions due to a loss of crossing fibers, and thus may lead to the hyper-excitability of the visual cortex. It is thought that the increased excitability of the visual cortex predisposes the brain to develop spontaneous neuronal depolarization, which may lead to cortical spreading depression (CSD), triggering headaches [47]. The CSD is a wave of slowly propagating depolarization followed by sustained suppression of neural activity, and has been thought to be the underlying mechanism of migraine [48, 49]. Therefore, we speculate that the impaired inferior fronto-occipital fasciculus may increase the vulnerability of CSD and lead to an increased risk of migraine.

The risk of migraine was also elevated by decreased OD of the posterior thalamic radiation, a part of posterior cortico-thalamic structural connectivity that connects the thalamus with the occipital and parietal cortex [50]. The interruption of thalamo-cortico-thalamic connectivity is responsible for multisensory integration dysfunction and thus thought as a potential source of clinical migraine symptoms [51]. A previous study has demonstrated impaired WM tracts located in the posterior thalamic radiation in migraine patients [52]. Moreover, neuroimaging studies using fMRI showed impaired functional connectivity between the posterior thalamus and occipital lobe, and their associations with patients’ clinical symptoms [53, 54]. Previous studies have confirmed the importance of posterior thalamus-occipital functional and structural connectivity in migraine pathophysiology. The current study extended these findings by identifying the causal effect of the posterior thalamic radiation on migraine. It is plausible that the posterior thalamic radiation collaborates with inferior fronto-occipital fasciculus to increase the vulnerability of CSD. Except for direct structural connectivity, the interactions between cortical regions also depend on the thalamus that synchronizes distant cortical regions via cortico-thalamic structural connectivity [55, 56]. Different from the inferior fronto-occipital fasciculus, decreased OD of the posterior thalamic radiation may impede indirect information flow from the occipital lobe to distant cortical regions mediated by the thalamus, resulting in a higher vulnerability of CSD.

Two WM IDPs were identified in our reverse MR analysis. Migraine leads to decreased OD of left superior cerebellar peduncle and decreased MO of right uncinate fasciculus. The superior cerebellar peduncle contains the main efferent fibers of the cerebellum [57]. It integrates the information from the cerebellum to thalamus that subsequently relays the information to cortical regions [58, 59]. According to previous studies, this tract was mainly involved in sensorimotor control and showed impairments in individuals with motor dysfunction [60, 61]. The OD is a parameter of NODDI that estimates the degree of neurite fiber dispersion [62]. Therefore, decreased OD indicated that migraine may impair the synaptic plasticity of the superior cerebellar peduncle. This putative causal effect could partly explain sensory and motor disturbances observed in migraine. Meanwhile, we also found significant causal associations between migraine and the uncinate fasciculus. This tract connects the amygdala to ventral prefrontal cortex and plays an important role in emotion regulation [63]. The disruption in this tract may contribute to common emotional dysfunction in migraine. However, this association should be carefully considered. A key assumption of MR analysis is that the IVs should act on the target outcome exclusively through the exposure of interest [39]. A violation of this assumption will reduce the credibility of MR results. The results of our sensitivity analyses, including MR-PRESSO Global test and Cochran’s Q statistic, suggested that the causal effects of migraine on the MO of right uncinate fasciculus might be inflated by some unknown confounders that violated this key assumption. In addition, the inference would not be robust if a causal association was dominated by a single SNP. Therefore, we further performed LOO analysis on the causal estimates and demonstrated that the MR results were not driven by any single SNP alone. These sensitivity analyses jointly improved the reliability of the MR results.

Overall, the present study identified several WM microstructures that can increase the risks of migraine or be altered by migraine. The WM IDPs identified by forward MR analysis may be potential neuroimaging biomarkers that can inform an individual of an elevated risk of migraine. The WM IDPs identified by reverse MR analysis provide potential intervention targets for migraine. Though our MR analysis cannot fully substitute for the randomized controlled trial evaluating intervention effects, it provides a guide for the design of future costly experiments.

The following limitations need to be considered. First, all samples of the selected GWAS in our study were of European ancestry. Though it reduced the bias induced by population stratification, to some extent, the generalizability of our findings might be limited. Second, the difference in sample size between the GWAS of migraine and IDPs gave rise to different power between forward and reverse MR analysis. The small sample size of IDPs GWAS may result in a lower power in reverse MR analysis rather than forward analysis [64]. In addition, fewer significant SNP IVs in the forward MR analysis may be accompanied by low power due to a limited explained proportion for total variation. To overcome insufficient power on weak associations, a GWAS of WM IDPs with a larger sample size is desired in the future. Third, the causal associations between WM IDPs and migraine subtypes (e.g., migraine with or without aura) were not further explored due to lack of the available GWAS with enough power (no more than 10,000 migraine cases at present) for MR analysis [65]. Finally, the associations between migraine symptoms and the identified WM IDPs are unclear. Future studies need to investigate the relationships between WM IDPs and key symptoms such as headache intensity and frequency.

## Conclusions

In conclusion, we identified the causal relationships between migraine and microstructural WM measured by IDPs of dMRI, based on genetic variants and bidirectional two-sample MR analysis. The findings provide a further understanding of neuropathophysiology underlying the development and experience of migraine, shedding light on potential targets for more precise and effective intervention on migraine.

## Supplementary Information

**Additional file 1: Appendix S1.** WM IDPs. Detailed description on 360 WM IDPs included in the present study. **Appendix S2.** Genetic correlation. The results of genetic correlation between WM IDPs and migraine. **Appendix S3.** Members of the IHGC. The members of the International Headache Genetics Consortium.

## Data Availability

The GWAS summary statistics of WM IDPs can be obtained from https://open.win.ox.ac.uk/ukbiobank/big40. The GWAS summary statistics of migraine are available by contacting the authors of the corresponding study.

## Abbreviations

WM: White matter
IDPs: Imaging-derived phenotypes
fMRI: Functional magnetic resonance imaging
sMRI: Structural magnetic resonance imaging
dMRI: Diffusion-weighted magnetic resonance imaging
DTI: Diffusion tensor imaging
NODDI: Neurite Orientation Dispersion and Density Imaging
ICV: Intracranial volume
TBSS: Tract-based spatial statistics
IVs: Instrumental variables
MR: Mendelian randomization
GWAS: Genome-wide association study
FA: Fractional anisotropy
MO: Mode of anisotropy
MD: Mean diffusivity
ICVF: Intracellular volume fraction
OD: Orientation dispersion index
ISOVF: Isotropic or free water volume fraction
SNPs: Single Nucleotide Polymorphisms
MAF: Minor allele frequency
LD: Linkage disequilibrium
IVW: Inverse-variance weighted
LOO: Leave-one-out
OR: Odds ratio
CI: Confidence interval
SD: Standard deviation
RSS: Residual sum of squares
CSD: Cortical spreading depression
